# Conjoint association of gestational diabetes mellitus and hypertensive disorders of pregnancy with long-term risk of mortality: a population-based cohort study

**DOI:** 10.7189/jogh.16.04204

**Published:** 2026-07-03

**Authors:** Darui Gao, Yang Zhang, Ying Gao, Jing Li, Chenglong Li, Jie Liang, Yang Pan, Wenya Zhang, Yongqian Wang, Mengmeng Ji, Junqing Zhang, Fanfan Zheng, Wuxiang Xie

**Affiliations:** 1 Department of Endocrinology, Peking University First Hospital, Beijing, China; 2 Clinical Research Institute, Institute of Advanced Clinical Medicine, Peking University, Beijing, China; 3 Key Laboratory of Epidemiology of Major Diseases, Peking University, Ministry of Education, Beijing, China; 4 School of Nursing, Peking Union Medical College, Chinese Academy of Medical Sciences, Beijing, China; 5 National Institute of Health Data Science at Peking University, Beijing, China

## Abstract

**Background:**

Gestational diabetes mellitus (GDM) and hypertensive disorders of pregnancy (HDPs) are common maternal complications. We sought to evaluate the independent and joint association of GDM and HDPs with all-cause, premature, and cause-specific mortality.

**Methods:**

We used data from the UK Biobank, which included 220 953 women who reported at least one live birth. Individual and joint associations of GDM and HDPs with all-cause, premature, and cause-specific mortality were estimated using Cox regression models.

**Results:**

During a follow-up of 12.9 years, women who experienced GDM had a higher risk of all-cause (hazard ratio (HR) = 1.57; 95% confidence interval (CI) = 1.26–1.96), premature (HR = 1.60; 95% CI = 1.24–2.06) and cardiovascular disease (CVD) mortality (HR = 2.60; 95% CI = 1.70–3.96). Women with a history of HDPs had an increased risk for CVD mortality (HR = 2.07; 95% CI = 1.51–2.83), but the association with all-cause death and premature death was not significant. Individuals who encountered both GDM and HDPs had a 3.9-fold higher relative risk of all-cause mortality (HR = 3.93; 95% CI = 1.88–8.24]) and a 4.3-fold higher risk of premature mortality (HR = 4.31; 95% CI = 1.94–9.57), compared to those without GDM or HDPs.

**Conclusions:**

Women with GDM have a higher risk of all-cause, premature mortality and both risks were higher than the impacts of a history of HDPs based on a large-scale population. The co-occurrence of GDM and HDPs will deteriorate the aforementioned situation compared to a single cardiometabolic pregnancy complication.

Gestational diabetes mellitus (GDM) and hypertensive disorders of pregnancy (HDPs) are the most common maternal complications during pregnancy, which are also known as cardiometabolic pregnancy complications, affecting up to 11.7–30.2% and 5.2–8.2% of pregnancies, respectively [1,2]. Given the overlapping physiological mechanisms, GDM and HDPs can sometimes co-occur. Previous studies have shown that GDM and HDPs can lead to various obstetric complications, including preterm birth, placental abruption, postpartum haemorrhage, and even maternal death [3]. Additionally, pregnancy has been referred to as a window to future maternal health, and the influence of GDM and HDPs extends beyond pregnancy. In recent years, accumulating evidence demonstrates that GDM and HDPs have long-term implications beyond pregnancy, including type two diabetes, cardiovascular disease (CVD), and cerebrovascular diseases in the years after pregnancy [4–7]. Furthermore, the co-occurrence of GDM and HDPs was associated with a much greater postpartum CVD, heart failure, and diabetes mellitus risk [8–10].

However, in addition to the long-term impact of cardiometabolic pregnancy complications on non-communicated diseases mentioned above, there remains a scarcity of studies that have explored the association between GDM or HDPs and hard endpoint mortality, which is unfavourable for identifying key lethal diseases in the long-term impact of GDM and HDPs. Previous observational studies related to GDM and mortality have mainly focused on maternal perinatal and in-hospital death or neonatal death [11–13]. Only one recent study reported an association between self-reported GDM and mortality among 91 426 female nurses in the USA [14]. Compared with GDM, the association between HDPs and mortality appears to have been studied more extensively, but the findings are inconsistent [15–17]. To date, only one study has attempted to examine the conjoint association of GDM and gestational hypertension (GH) with mortality, indicating that a history of both GDM and GH resulted in a hazard ratio (HR) for the composite CVD and mortality of 2.4 (95% confidence interval (CI) = 1.2–1.7), higher than a history of either GDM or GH [18]. However, this study defined a composite outcome of CVD and mortality instead of including mortality-related outcomes separately from the composite outcome, which is not conducive to elucidating the specific causes of death other than CVD for such pregnancy complications in long-term postpartum follow-up.

Considering the above, a comprehensive assessment of the long-term mortality risk of GDM and/or HDPs, including all-cause death, premature death, and corresponding causes of death, will greatly understand the fatal related diseases of these women and might help to identify prevention and intervention targets earlier, which is of great significance for improving the overall life expectancy of the population. Using data from the UK Biobank, we aimed to examine the association between independent GDM or HDPs and the combined impact of these on all-cause, premature, and cause-specific mortality, to thoroughly explore the association between cardiometabolic pregnancy complications and long-term postpartum mortality.

## METHODS

### Study population

We based this study on a publicly available large-scale population database, and we fully consent to and support the policy of GRABDROP guidelines (Table S1 in the **Online Supplementary Document**). The UK Biobank includes over 500 000 volunteers aged 40–69 years at baseline recruited through the UK National Health Service registers during 2006–2010 [19]. Participants included those who attended assessment visits comprising electronically signed consent, self-completed touchscreen questionnaires, brief computer-assisted interviews, physical and functional measures, and who provided biological samples in 22 assessment centres throughout the UK [20]. The UK Biobank study was approved by the National Information Governance Board for Health and Social Care and the National Health Service North West Multi-Centre Research Ethics Committee [16]. All participants provided electronically signed consent.

Among 502 411 participants from UK Biobank, we included women who reported at least one live birth and had known parity information. We excluded participants if they were male (n = 229 086), nulliparous at baseline (n = 51 089), lacked parity data (n = 830) or had first pregnancy information data (n = 453). After these exclusions, an analytic cohort of 220 953 participants was yielded (Figure S1 in the **Online Supplementary Document**).

### Exposure assessment

GDM was defined by self-reports from participants at enrolment or by the International Classification of Diseases (ICD) codes. All the female participants were asked whether they had a history of GDM during pregnancy by touchscreen questionnaire or verbal interview. In addition, the UK Biobank collected data on disease diagnoses recorded before enrolment and during follow-up, and GDM was defined using ICD-10 code O24. Similarly, HDPs were ascertained using self-reported data and ICD-10 codes. In self-reported data, HDPs were determined from those who self-reported having gestational hypertension/pre-eclampsia. Based on previous studies, the ICD-10 codes used to identify HDPs were O10 (pre-existing hypertension complicating pregnancy, childbirth and the puerperium), O11 (pre-existing hypertensive disorder with superimposed proteinuria), O13 (gestational [pregnancy-induced] hypertension without significant proteinuria), O14 (gestational [pregnancy-induced] hypertension with significant proteinuria), O15 (eclampsia) and O16 (unspecified maternal hypertension) [21].

### Ascertainment of outcomes

We obtained the date and cause of death from death certificates held by the National Health Service Information Centre (England and Wales) and the Central Register of Scotland (Scotland). The causes of death (cancer, CVD, respiratory disease, digestive disease, neurodegenerative disease, and other causes) were defined based on ICD-10 codes (Table S2 in the **Online Supplementary Document**) [22]. The primary outcome of this study was all-cause mortality. Additionally, premature mortality, defined as death that occurred at ages <75 years, was the secondary outcome [23].

### Covariates

Covariates included socio-demographics, behavioural factors and clinical characteristics. Socio-demographics included race and educational status. Behavioural factors included current smoking, alcohol intake and physical activity. Clinical characteristics included age at first live birth (years), multiple live births (number of live births ≥5 times), obesity (defined according to body mass index (BMI)≥30 kg/m^2^ at enrolment), depressed mood and hyperlipidaemia at baseline. We additionally adjusted for hypertension at baseline when the exposure variable was GDM. And when the exposure was HDPs, baseline diabetes was additionally adjusted as a covariate (Table S3 in the **Online Supplementary Document**).

### Statistical analysis

For descriptive statistics, variables were reported as numbers and percentages for categorical variables, mean and standard deviation (SD) for normally distributed variables, and median and interquartile range (IQR) for nonnormally distributed variables. The baseline characteristics of the study population were presented by categories of GDM and HDPs, including four groups: no GDM and no-HDPs, GDM and no-HDPs, no GDM and HDPs, and GDM and HDPs. We tested the differences in baseline characteristics using the χ^2^ test for categorical variables, analysis of variance for normally distributed continuous variables, and the Kruskal-Wallis test for nonnormally distributed continuous variables. However, it should be noted that these tests would be significant even for small differences, given the large sample size [22].

We computed the follow-up time from the date of attending baseline assessment until the date of death or the end of follow-up (31 December 2021), whichever came first. We first examined the independent associations of a history of GDM (with no GDM as the reference) or HDPs (with no HDPs as the reference) with all-cause, premature, and cause-specific mortality risk using Cox proportional hazards models, with age as the time scale variable. We presented the results as HRs with 95% CI. To quantify the burden of mortality incidence attributable to GDM and HDPs separately, we calculated a multivariable-adjusted population attributable fraction (PAF) using the model-based approach for a time-to-event outcome [24] (Methods S1 in the **Online Supplementary Document**). Furthermore, to investigate potential modifying effects of covariates on the association between independent GDM and HDPs and mortality, we used the Z-test proposed by Altman and Bland to compare the two regression coefficients from each subgroup analysis [25].

We also examined the joint association between GDM and HDPs with all-cause and premature mortality using Cox proportional hazards models, with age as the time scale variable. We used the group with neither GDM history nor HDP history as a reference. We first compared outcomes among the study groups using the log-rank test with Kaplan-Meier curves. Then, we conducted sequential adjustments. Analyses started with a minimally adjusted Cox model including only age at first live birth, multiple live births, race, and educational status (adjusted model 1), and a second model additionally adjusted for obesity, current smoking, alcohol intake, physical activity, depressed mood and hyperlipidaemia at baseline (adjusted model 2). To further examine the potential synergistic effect between GDM and HDPs, a product interaction term (GDM × HDPs) was included in the Cox proportional hazards models for all-cause and premature mortality. Statistical significance of the interaction term was assessed using Wald tests [26].

### Sensitivity analyses

We performed several sensitivity analyses to test the robustness of our major findings. We excluded participants who died or lost follow-up within five years after baseline to further control possible reverse causality. We excluded participants aged ≤60 years at baseline because mortality risk may be relatively low. Given the unprecedented impact of the COVID-19 pandemic on healthcare systems, we restricted our follow-up to the pre-pandemic period by 31 December 2019.

We performed all analyses using SAS, version 9.4 (SAS Institute, Cary, North Carolina, USA) and *R*, version 4.3.0 (R Core Team, Vienna, Austria). Statistical significance was defined as *P* < 0.05 and all tests were two-tailed.

## RESULTS

### Basic characteristics of the study population

The study sample included 220 953 women, with a mean age of 57.4 years (SD = 7.9 years). Among them, 1331 women had isolated GDM (0.60%), 3810 women had isolated HDPs (1.72%), and 123 women had a combination of GDM and HDPs (0.06%). Women with a combination GDM and HDPs were younger at enrolment, more likely to had a higher educational status and older at the first live birth; they were less likely to have a healthy lifestyle with higher rates of obesity, smoking and lower rate of keeping exercising; and had higher prevalence of depression mood, hyperlipidaemia, diabetes and hypertension at enrolment (Table S4 in the **Online Supplementary Document**).

### Independent associations of exposures with mortality

During a median follow-up of 12.9 years (IQR = 12.2–13.6), 12 508 and 8787 incident cases of all-cause and premature death were documented, respectively. A history of GDM was associated with higher risks of all-cause and premature mortality in our multivariable-adjusted model, with adjusted HRs of 1.57 (95% CI = 1.26–1.96) for all-cause mortality and 1.60 (95% CI = 1.24–2.06) for premature mortality. However, no significant associations were found between a history of HDPs and all-cause and premature mortality (**Table 1**).

**Table 1:** History of gestational diabetes mellitus and hypertensive disorders of pregnancy and the risk of all-cause and premature mortality

Exposures	n/N	HR (95% CI)	*P*-value*
**All-cause mortality as an event**			
Gestational diabetes mellitus			
*No*	12 429/219 499	ref	
*Yes*	79/1454	1.57 (1.26–1.96)	<0.001
Hypertensive disorders of pregnancy			
*No*	12 357/217 020	ref	
*Yes*	151/3933	1.16 (0.99–1.37)	0.066
**Premature mortality as an event**			
Gestational diabetes mellitus			
*No*	8726/219 499	ref	
*Yes*	61/1454	1.60 (1.24–2.06)	<0.001
Hypertensive disorders of pregnancy			
*No*	8682/217 020	ref	
*Yes*	105/3933	1.10 (0.91–1.34)	0.313

CI – confidence interval, HR – hazard ratio, ref – reference

*When gestational diabetes mellitus was the exposure, *P*-values were adjusted for age at first live birth, multiple live births, race, educational status, obesity, current smoking, alcohol intake, physical activity, depressed mood, hyperlipidaemia, and hypertension at baseline. When hypertensive disorders of pregnancy were exposed, *P*-values were adjusted for age at first live birth, multiple live births, race, educational status, obesity, current smoking, alcohol intake, physical activity, depressed mood, hyperlipidaemia, and diabetes at baseline.

### Joint association of exposures with mortality

**Figure 1:**
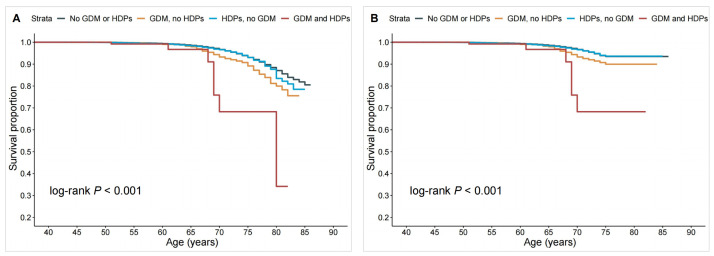
Kaplan-Meier curves for all-cause and premature mortality by gestational diabetes mellitus (GDM) and hypertensive disorders of pregnancy (HDPs) status. **Panel A.** Kaplan-Meier curves for all-cause mortality. **Panel B.** Kaplan-Meier curves for premature mortality.

Individuals who encountered both GDM and HDPs exhibited the highest incidence of all-cause and premature mortality among all study groups (**Figure 1**, **Table 2**). They had a 3.9-fold higher relative risk of all-cause mortality (HR = 3.93; 95% CI = 1.88–8.24) in the fully adjusted model and a 4.3-fold higher risk of premature mortality (adjusted HR (aHR) = 4.31; 95% CI = 1.94–9.57), compared to those without GDM or HDPs. However, due to the small number of women in this subgroup (n = 123), these estimates should be interpreted with caution. The test for multiplicative interaction revealed a significant synergistic effect between GDM and HDPs for both all-cause mortality (*P* = 0.037) and premature mortality (*P* = 0.025), suggesting that the co-occurrence of these conditions may confer excess mortality risk beyond their independent effects.

**Table 2:** Event rates and hazard ratios for the association of gestational diabetes mellitus and hypertensive disorders of pregnancy with all-cause and premature mortality

Exposures	All-cause mortality			
	**Crude IR per 1000 PY (95% CI)**	**HR (95% CI)**		
		**Unadjusted model**	Model 1*	**Model 2**†
All-cause mortality as an event				
*No HDPs and no GDM*	8.11 (7.99–8.23)	ref	ref	Ref.
*Isolated GDM*	8.27 (6.81–10.04)	1.64 (1.30–2.07)	1.65 (1.31–2.08)	1.49 (1.18–1.88)
*Isolated HDPs*	5.83 (5.08–6.68)	1.11 (0.95–1.31)	1.14 (0.97–1.35)	1.14 (0.96–1.34)
*HDPs and GDM*	9.50 (5.10–17.68)	4.44 (2.12–9.33)	4.78 (2.28–10.03)	3.93 (1.88–8.24)
**Premature mortality as an event**				
*No HDPs and no GDM*	5.70 (5.60–5.80)	ref	ref	Ref.
*Isolated GDM*	6.32 (5.06–7.89)	1.64 (1.25–2.13)	1.64 (1.26–2.14)	1.50 (1.15–1.96)
*Isolated HDPs*	4.01 (3.40–4.73)	1.03 (0.85–1.26)	1.06 (0.88–1.29)	1.07 (0.88–1.30)
*HDPs and GDM*	8.14 (4.16–15.93)	4.63 (2.08–10.29)	4.97 (2.24–11.04)	4.31 (1.94–9.57)

CI – confidence interval, GDM – gestational diabetes mellitus, HDPs – hypertensive disorders of pregnancy, HR – hazard ratio, IR – incidence rate, PY – person-years, ref – reference

*Model 1: adjusted for age at first live birth, multiple live births, race, and educational status.

†Model 2: model 1 plus obesity, current smoking, alcohol intake, physical activity, depressed mood and hyperlipidaemia at baseline.

### Death attribution analysis of GDM and HDPs

For cause-specific mortality, adjusted HRs for participants with a history of GDM were 2.60 (95% CI = 91.70–3.96) for CVD mortality and 2.34 (95% CI = 1.41–3.90) for other mortality (death from other causes other than cancer, CVD, respiratory disease, digestive disease, and neurodegenerative disease), relative to women without a GDM history (**Table 3**). Similarly, HDPs were also associated with higher mortality risk of CVD (aHR = 2.07; 95% CI = 1.51–2.83) and other (aHR = 1.69; 95% CI = 1.14–2.52).

**Table 3:** HRs (95% CIs) for cause-specific mortality of gestational diabetes mellitus and hypertensive disorders of pregnancy

Cause of death	Gestational diabetes mellitus			Hypertensive disorders of pregnancy		
	**n**	**HR (95% CI)**	*P*-value*	**n**	**HR (95% CI)**	***P-v*alue**†
Cancer	30	1.10 (0.77–1.57)	0.606	63	0.84 (0.66–1.08)	0.174
Cardiovascular disease	22	2.60 (1.70–3.96)	<0.001	40	2.07 (1.51–2.83)	<0.001
Respiratory disease	4	1.16 (0.43–3.10)	0.771	8	1.05 (0.52–2.10)	0.899
Digestive disease	2	1.05 (0.26–4.21)	0.949	3	0.68 (0.22–2.13)	0.510
Neurodegenerative disease	6	1.95 (0.87–4.35)	0.104	12	1.34 (0.76–2.36)	0.318
Other	15	2.34 (1.41–3.90)	0.001	25	1.69 (1.14–2.52)	0.009

CI – confidence interval, HR – hazard ratio

*When gestational diabetes mellitus was the exposure, *P*-values were adjusted for age at first live birth, multiple live births, race, educational status, obesity, current smoking, alcohol intake, physical activity, depressed mood, hyperlipidaemia, and hypertension at baseline.

†When hypertensive disorders of pregnancy as exposure, P value were adjusted for age at first live birth, multiple live births, race, educational status, obesity, current smoking, alcohol intake, physical activity, depressed mood, hyperlipidaemia, and type two diabetes at baseline.

GDM was an attributable exposure of all-cause and CVD mortality, with PAF of 0.32–0.41% and 1.17–1.30%, respectively. While PAF for CVD mortality of HDPs exposure was 1.70–1.80% (Figures S2 and S3 in the **Online Supplementary Document**).

In our subgroup analyses, an interesting finding was that multiple live births modified the association between HDPs and incident CVD mortality (*P* < 0.001). Specifically, we found that this association turned out to be stronger in women with a number of live births ≥5 times (HR = 11.76; 95% CI = 4.92–28.10 *vs.* HR = 1.81; 95% CI = 1.29–2.55) (Figures S4–6 in the **Online Supplementary Document**).

### Sensitivity analysis

Generally, similar findings were seen in the sensitivity analysis excluding patients who died or lost follow-up within five years after baseline. Little differences were observed in the two other sensitivity analyses (excluding patients aged ≤60 years old at baseline or further restricting to the pre-pandemic follow-up period) with multiplicative interactions not statistically significant (Tables S5–7 in the **Online Supplementary Document**). However, the HRs of the HDPs and GDM combination group were the highest among the study groups and statistically significant.

## DISCUSSION

In this large-scale prospective cohort study, we had three main important findings: a history of GDM was significantly associated with a higher hazard of all-cause, premature and CVD mortality; an increased risk of death from CVD was observed in women with HDPs compared to those without, though the associations between HDPs with all-cause and premature mortality were not significant; and women who experienced both GDM and HDPs during pregnancy were ultimately at significantly higher absolute and relative risk of all-cause and premature mortality, as compared to those without either of these conditions, and this findings merits careful consideration.

The independent association of GDM or HDPs history with CVD, type two diabetes and cerebrovascular diseases during long-term follow-up postpartum has been extensively documented, suggesting their link to elevated mortality risk [4–6,16,27]. To date, however, there remains a scarcity of long-term cohort studies that have examined the specific association between GDM and HDPs and mortality (particularly for GDM). Previous studies of GDM and mortality mainly focused on the risk of maternal cardiac arrest and in-hospital death, and adverse foetal mortality outcomes [28,29]. Only one recent study has reported that GDM was associated with all-cause death with an HR of 1.25 (95% CI = 1.11–1.41) [14]. However, this study was conducted on a representative, limited sample (all enrolled participants were female nurses in the USA). Our study further identified GDM as an important risk factor for all-cause, premature, and CVD mortality using data from a community-based sample. Notably, according to our findings, GDM appeared to be associated with a higher risk of long-term all-cause and premature mortality than HDPs. In recent years, several long-term observational studies reported that HDPs were associated with an increased risk of CVD-related mortality, which were consistent with our findings [15,16,30,31]. However, previous evidence on the association of HDPs with all-cause or premature mortality was inconsistent. Of these, results from two population-based studies in the United States showed a positive and significant association [15,16]. On the contrary, a cohort study involving 3 593 women from Scotland found no association between gestational hypertension and all-cause mortality (HR = 1.03; 95% CI = 0.74–1.45), which was in line with our present study [17].

To the best of our knowledge, this is the first study to investigate the long-term joint association between GDM and HDPs and mortality in a large population. Previous studies have mainly focused on the association between combined pregnancy complications and long-term cardiovascular disease. Similar to our study design, a recent study reported a significantly higher risk of CVD among women who experienced both GDM and HDPs but did not further explore the CVD mortality [8]. In support of our findings, a previous study suggested that a history of both GDM and HDPs increased the HR to 2.4 for the composite CVD and mortality outcome [18]. Considering that the long-term impact of GDM and HDPs is not limited to CVD, metabolic diseases, tumours, and psychiatric disorders have also been reported to be associated with the aforementioned maternal pregnancy complications, it is necessary to fully evaluate the long-term mortality risk of GDM and HDPs and analyse the cause of death to identify key fatal events. Our work extends the two Canadian cohort studies above, which did not treat all-cause death and premature death as separate outcome indicators in the analysis. In addition, we further investigated the specific causes of death other than CVD.

Although the precise physiological mechanisms of the joint association of GDM and HDPs with a higher risk of mortality remain to be determined, our current findings sound plausible in consideration of several possible explanations. The first explanation is that GDM and HDPs share similar pathophysiologic abnormalities [3,32,33]. Both GDM and HDPs have been linked to insulin resistance and an up-regulated innate immune response, which, in turn, is associated with inflammation, vascular dysfunction, oxidative stress and vascular disease [32,34,35]. These shared pathophysiologic abnormalities might have a stronger negative impact on certain end target organs. Although GDM and HDPs are always transient and resolve after giving birth, these abnormalities might leave an imprint that may further increase the risk of mortality over a long period in later life [29,36–38]. Another possible explanation regarding the common risk factors among GDM, HDPs and mortality. As observed in our present study, individuals who experienced both GDM and HDPs had higher BMI, older age at first live birth, and were less likely to continue exercising, all of which are well-established risk factors for all-cause mortality and thus collectively increased the burden of mortality [39–42].

The present findings may have several clinical and public health implications. The long-term risk of death observed after GDM further confirms that, in addition to HDPs, GDM also has a long-term postpartum adverse impact that cannot be ignored. In addition, the long-term mortality rate of women with a history of GDM combined with HDPs is much higher than that of any single complication, and CVD is the main cause of death in women with a history of cardiometabolic pregnancy complications. Taking this evidence together, we believe that continuous postpartum monitoring and long-term appropriate strategies for preventing CVD may reduce mortality and prolong life expectancy in women who reported a history of GDM or HDPs [25], and the urgency escalates when both conditions coexist. Except for traditional CVD prevention and intervention, results from our subgroup analysis indicated that a reasonable number of births may be beneficial to reduce the disease burden associated with HDPs.

The strengths of our study include the prospective cohort design, long follow-up, large sample size and rich data resources, which enable detailed analyses on mortality from multiple causes. Several potential limitations should be carefully considered in the present study. First, given the nature of the UK Biobank, our study should be considered a retrospective cohort study rather than a prospective one, as the exposures occurred before baseline enrolment. This design limits our ability to establish temporal relationships and infer causality. Second, 52 372 female participants were excluded, potentially introducing selection bias. Comparison of baseline characteristics between participants included (n = 220 953) and excluded demonstrated a significant difference (Table S8 in the **Online Supplementary Document**). In general, the participants excluded were younger and more highly educated, which may bias the associations observed in this study. Third, participants in the UK Biobank are not a representative population-based sample. They are primarily White and were recruited via volunteer sampling without systematic selection. In addition, they tend to be healthier than the general population (*i.e.* ‘healthy volunteer’ selection bias). These factors collectively limit the generalisability of our findings to other ethnic groups and broader populations. Therefore, further validations in other populations are warranted. Fourth, given the observational nature of the data, residual confounding was inevitable, and a causal relationship cannot be concluded. Fifth, it is important to acknowledge that the wide confidence intervals for the combined GDM and HDPs group reflect the small number of women with both conditions, which limits the precision of the risk estimates. Future studies with larger sample sizes are needed to confirm these findings and further explore the potential synergistic mechanisms underlying the joint effect of GDM and HDPs on long-term mortality. Sixth, there was a substantial time interval between pregnancy and baseline enrolment. Women who died from pregnancy-related complications or other causes during this interval were not captured in the UK Biobank, introducing survival bias. This likely leads to an underestimation of the true mortality risks associated with GDM and HDPs. Furthermore, our sensitivity analysis excluding women younger than 60 years may have compounded this bias by further restricting the sample to longer-term survivors. Seventh, the prevalence of GDM and HDPs in our study sample was substantially lower than the modern population rates reported in the literature. This underascertainment likely reflects several factors: recall bias due to self-reporting of exposures decades after pregnancy; the ‘healthy volunteer’ bias inherent to the UK Biobank; and changes in diagnostic practices over time. Notably, universal screening for GDM did not become routine obstetric practice until the late 1990s [43], and before this era, the reported prevalence of GDM was only 1–2% [44]. As the majority of women in our cohort (86.6%) had their first live birth before the 1990s, the low self-reported prevalence observed is consistent with the diagnostic standards of that period [45]. Consequently, many women with milder or undiagnosed GDM or HDPs may have been misclassified into the non-exposed group.

## CONCLUSIONS

Our study indicates for the first time that women with a history of GDM have a higher risk of all-cause, premature mortality and this risk was both higher than that of a history of HDPs based on a national population. And the co-occurrence of GDM and HDPs will deteriorate the aforementioned situation compared to a single cardiometabolic pregnancy complication. Our results support the value of long-term monitoring and interventions for chronic non-communicable diseases with a focus on CVD for women with histories of GDM and HDPs to improve health and prolong life expectancy. However, due to the retrospective nature of the exposure assessment and the selection bias inherent in the UK Biobank, these findings should be interpreted with caution and validated in more representative cohorts.

## Additional material


Online Supplementary Document

## References

[1] Umesawa M Kobashi G Epidemiology of hypertensive disorders in pregnancy: prevalence, risk factors, predictors and prognosis. Hypertens Res. 2017;40:213–20. 10.1038/hr.2016.126 27682655

[2] Wang H Li N Chivese T Werfalli M Sun H Yuen L IDF Diabetes Atlas: Estimation of Global and Regional Gestational Diabetes Mellitus Prevalence for 2021 by International Association of Diabetes in Pregnancy Study Group’s Criteria. Diabetes Res Clin Pract. 2022;183:109050. 10.1016/j.diabres.2021.109050 34883186

[3] Jiang L Tang K Magee LA von Dadelszen P Ekeroma A Li X A global view of hypertensive disorders and diabetes mellitus during pregnancy. Nat Rev Endocrinol. 2022;18:760–75. 10.1038/s41574-022-00734-y 36109676 PMC9483536

[4] Grandi SM Filion KB Yoon S Ayele HT Doyle CM Hutcheon JA Cardiovascular Disease-Related Morbidity and Mortality in Women With a History of Pregnancy Complications. Circulation. 2019;139:1069–79. 10.1161/CIRCULATIONAHA.118.036748 30779636

[5] Honigberg MC Zekavat SM Aragam K Klarin D Bhatt DL Scott NS Long-Term Cardiovascular Risk in Women With Hypertension During Pregnancy. J Am Coll Cardiol. 2019;74:2743–54. 10.1016/j.jacc.2019.09.052 31727424 PMC6981240

[6] Lee SM Shivakumar M Park JW Jung YM Choe EK Kwak SH Long-term cardiovascular outcomes of gestational diabetes mellitus: a prospective UK Biobank study. Cardiovasc Diabetol. 2022;21:221. 10.1186/s12933-022-01663-w 36309714 PMC9618212

[7] Xie W Wang Y Xiao S Qiu L Yu Y Zhang Z Association of gestational diabetes mellitus with overall and type specific cardiovascular and cerebrovascular diseases: systematic review and meta-analysis. BMJ. 2022;378:e070244. 10.1136/bmj-2022-070244 36130740 PMC9490552

[8] Echouffo Tcheugui JB Guan J Fu L Retnakaran R Shah BR Association of Concomitant Gestational Hypertensive Disorders and Gestational Diabetes With Cardiovascular Disease. JAMA Netw Open. 2022;5:e2243618. 10.1001/jamanetworkopen.2022.43618 36416822 PMC9685489

[9] Echouffo-Tcheugui JB Guan J Fu L Retnakaran R Shah BR Incidence of Heart Failure Related to Co-Occurrence of Gestational Hypertensive Disorders and Gestational Diabetes. JACC Adv. 2023;2:100377. 10.1016/j.jacadv.2023.100377 37476567 PMC10358333

[10] Kek HP Su YT Tey SJ Yang MC Chang LC Hung YH The joint effect of gestational diabetes mellitus and hypertension contribute to higher risk of diabetes mellitus after delivery: a nationwide population-based study. BMC Pregnancy Childbirth. 2023;23:539. 10.1186/s12884-023-05829-6 37495968 PMC10373314

[11] Battarbee AN Venkatesh KK Aliaga S Boggess KA The association of pregestational and gestational diabetes with severe neonatal morbidity and mortality. J Perinatol. 2020;40:232–9. 10.1038/s41372-019-0516-5 31591489

[12] Morikawa M Sugiyama T Sagawa N Hiramatsu Y Ishikawa H Hamada H Perinatal mortality in Japanese women diagnosed with gestational diabetes mellitus and diabetes mellitus. J Obstet Gynaecol Res. 2017;43:1700–7. 10.1111/jog.13431 28817202

[13] Sunjaya AP Sunjaya AF Diabetes in pregnancy and infant mortality: Link with glycemic control. Diabetes Metab Syndr. 2018;12:1031–7. 10.1016/j.dsx.2018.06.019 29945772

[14] Wang YX Mitsunami M Manson JE Gaskins AJ Rich-Edwards JW Wang L Association of Gestational Diabetes With Subsequent Long-Term Risk of Mortality. JAMA Intern Med. 2023;183:1204–1213. 10.1001/jamainternmed.2023.4401 37695588 PMC10495928

[15] Theilen LH Fraser A Hollingshaus MS Schliep KC Varner MW Smith KR All-Cause and Cause-Specific Mortality After Hypertensive Disease of Pregnancy. Obstet Gynecol. 2016;128:238–44. 10.1097/AOG.0000000000001534 27400006 PMC4961555

[16] Wang YX Arvizu M Rich-Edwards JW Wang L Rosner B Stuart JJ Hypertensive Disorders of Pregnancy and Subsequent Risk of Premature Mortality. J Am Coll Cardiol. 2021;77:1302–12. 10.1016/j.jacc.2021.01.018 33706872 PMC7959184

[17] Wilson BJ Watson MS Prescott GJ Sunderland S Campbell DM Hannaford P Hypertensive diseases of pregnancy and risk of hypertension and stroke in later life: results from cohort study. BMJ. 2003;326:845. 10.1136/bmj.326.7394.845 12702615 PMC153466

[18] Pace R Brazeau AS Meltzer S Rahme E Dasgupta K Conjoint Associations of Gestational Diabetes and Hypertension With Diabetes, Hypertension, and Cardiovascular Disease in Parents: A Retrospective Cohort Study. Am J Epidemiol. 2017;186:1115–24. 10.1093/aje/kwx263 29149255 PMC5859985

[19] Sudlow C Gallacher J Allen N Beral V Burton P Danesh J UK biobank: an open access resource for identifying the causes of a wide range of complex diseases of middle and old age. PLoS Med. 2015;12:e1001779. 10.1371/journal.pmed.1001779 25826379 PMC4380465

[20] Ollier W Sprosen T Peakman TUK Biobank: from concept to reality. Pharmacogenomics. 2005;6:639–46. 10.2217/14622416.6.6.639 16143003

[21] Ford ND Cox S Ko JY Ouyang L Romero L Colarusso T Hypertensive Disorders in Pregnancy and Mortality at Delivery Hospitalization - United States, 2017-2019. MMWR Morb Mortal Wkly Rep. 2022;71:585–91. 10.15585/mmwr.mm7117a1 35482575 PMC9098235

[22] Han H Cao Y Feng C Zheng Y Dhana K Zhu S Association of a Healthy Lifestyle With All-Cause and Cause-Specific Mortality Among Individuals With Type 2 Diabetes: A Prospective Study in UK Biobank. Diabetes Care. 2022;45:319–29. 10.2337/dc21-1512 34857534

[23] Lewer D Jayatunga W Aldridge RW Edge C Marmot M Story A Premature mortality attributable to socioeconomic inequality in England between 2003 and 2018: an observational study. Lancet Public Health. 2020;5:e33–41. 10.1016/S2468-2667(19)30219-1 31813773 PMC7098478

[24] Chen L Lin DY Zeng D Attributable fraction functions for censored event times. Biometrika. 2010;97:713–26. 10.1093/biomet/asq023 23956459 PMC3744602

[25] Altman DG Bland JM Interaction revisited: the difference between two estimates. BMJ. 2003;326:219. 10.1136/bmj.326.7382.219 12543843 PMC1125071

[26] Xia PF Pan XF Chen C Wang Y Ye Y Pan A Dietary Intakes of Eggs and Cholesterol in Relation to All-Cause and Heart Disease Mortality: A Prospective Cohort Study. J Am Heart Assoc. 2020;9:e015743. 10.1161/JAHA.119.015743 32400247 PMC7660855

[27] Vounzoulaki E Khunti K Abner SC Tan BK Davies MJ Gillies CL Progression to type 2 diabetes in women with a known history of gestational diabetes: systematic review and meta-analysis. BMJ. 2020;369:m1361. 10.1136/bmj.m1361 32404325 PMC7218708

[28] Huang C Wei K Lee PMY Qin G Yu Y Li J Maternal hypertensive disorder of pregnancy and mortality in offspring from birth to young adulthood: national population based cohort study. BMJ. 2022;379:e072157. 10.1136/bmj-2022-072157 36261141 PMC9580246

[29] Tavera G Dongarwar D Salemi JL Akindela O Osazuwa I Akpan EB Diabetes in pregnancy and risk of near-miss, maternal mortality and foetal outcomes in the USA: a retrospective cross-sectional analysis. J Public Health (Oxf). 2022;44:549–57. 10.1093/pubmed/fdab117 33866358

[30] Mongraw-Chaffin ML Cirillo PM Cohn BA Preeclampsia and cardiovascular disease death: prospective evidence from the child health and development studies cohort. Hypertension. 2010;56:166–71. 10.1161/HYPERTENSIONAHA.110.150078 20516394 PMC3037281

[31] Skjaerven R Wilcox AJ Klungsøyr K Irgens LM Vikse BE Vatten LJ Cardiovascular mortality after pre-eclampsia in one child mothers: prospective, population based cohort study. BMJ. 2012;345:e7677. 10.1136/bmj.e7677 23186909 PMC3508198

[32] Sibai BM Ross MG Hypertension in gestational diabetes mellitus: pathophysiology and long-term consequences. J Matern Fetal Neonatal Med. 2010;23:229–33. 10.3109/14767050903550899 20121395

[33] Yang Y Wu N Gestational Diabetes Mellitus and Preeclampsia: Correlation and Influencing Factors. Front Cardiovasc Med. 2022;9:831297. 10.3389/fcvm.2022.831297 35252402 PMC8889031

[34] Carpenter MW Gestational diabetes, pregnancy hypertension, and late vascular disease. Diabetes Care. 2007;30:S246–50. 10.2337/dc07-s224 17596480

[35] Heitritter SM Solomon CG Mitchell GF Skali-Ounis N Seely EW Subclinical inflammation and vascular dysfunction in women with previous gestational diabetes mellitus. J Clin Endocrinol Metab. 2005;90:3983–8. 10.1210/jc.2004-2494 15840749

[36] Ives CW Sinkey R Rajapreyar I Tita ATN Oparil S Preeclampsia-Pathophysiology and Clinical Presentations: JACC State-of-the-Art Review. J Am Coll Cardiol. 2020;76:1690–702. 10.1016/j.jacc.2020.08.014 33004135

[37] Levine RJ Maynard SE Qian C Lim KH England LJ Yu KF Circulating angiogenic factors and the risk of preeclampsia. N Engl J Med. 2004;350:672–83. 10.1056/NEJMoa031884 14764923

[38] Martins IJ Diabetes and Organ Dysfunction in the Developing and Developed World. J Med Res. 2015;15:15-21.

[39] Feng H Yang L Liang YY Ai S Liu Y Liu Y Associations of timing of physical activity with all-cause and cause-specific mortality in a prospective cohort study. Nat Commun. 2023;14:930. 10.1038/s41467-023-36546-5 36805455 PMC9938683

[40] Flegal KM Kit BK Orpana H Graubard BI Association of all-cause mortality with overweight and obesity using standard body mass index categories: a systematic review and meta-analysis. JAMA. 2013;309:71–82. 10.1001/jama.2012.113905 23280227 PMC4855514

[41] Restrepo-Méndez MC Victora CG Maternal mortality by age: who is most at risk? Lancet Glob Health. 2014;2:e120–1. 10.1016/S2214-109X(14)70007-5 25102834

[42] Temmerman M Verstraelen H Martens G Bekaert A Delayed childbearing and maternal mortality. Eur J Obstet Gynecol Reprod Biol. 2004;114:19–22. 10.1016/j.ejogrb.2003.09.019 15099865

[43] Danilenko-Dixon DR Van Winter JT Nelson RL Ogburn PL Jr Universal versus selective gestational diabetes screening: application of 1997 American Diabetes Association recommendations. Am J Obstet Gynecol. 1999;181:798–802. 10.1016/S0002-9378(99)70304-2 10521732

[44] Ferrara A Increasing prevalence of gestational diabetes mellitus: a public health perspective. Diabetes Care. 2007;30:S141–6. 10.2337/dc07-s206 17596462

[45] Zhang Y Gao D Gao Y Li J Li C Pan Y Gestational diabetes mellitus is associated with greater incidence of dementia during long-term post-partum follow-up. J Intern Med. 2024;295:774–84. 10.1111/joim.13787 38629919

