# Supplementary material for: Conjoint association of gestational diabetes mellitus and hypertensive disorders of pregnancy with long-term risk of mortality: a population-based cohort study
Source: J Glob Health. 2026 Jul 3;16:04204. doi: 10.7189/jogh.16.04204 (PMC13329938; doi:10.7189/jogh.16.04204)
Supplement: Online Supplementary Document [file jogh-16-04204-s001.pdf]

Supplement to: Gao D, Zhang Y, Gao Y, Li J, Li C, Liang J, Pan Y, Zhang W, Wang Y, Ji M, Zhang J, Zheng F, Xie W. Conjoint association of gestational diabetes mellitus and hypertensive disorders of pregnancy with long-term risk of mortality: a population-based cohort study. J Glob Health. 2026;16:04204.

Figure S1. Participants selection diagram 2
Table S1. Outline of JoGH's Guidelines for Reporting Analyses of Big Data Repositories Open to the Public (GRABDROP) items 3
Table S2. International Classification of Diseases (ICD) codes used for identification of cause-specific mortality* 8
Table S3. Covariates definition and assessment 9
Supplement Methods 11
Table S4. Baseline characteristics of study participants by gestational diabetes mellitus (GDM) and/or hypertensive disorders of pregnancy (HDPs) status 12
Figure S2. Population attributable fraction of all-cause mortality 14
Figure S3. Population attributable fraction of cardiovascular disease mortality 15
Figure S4. Subgroup analyses to identify potential modifying effects on the association between gestational diabetes mellitus and all-cause mortality by using Cox proportional hazards models 16
Figure S5. Subgroup analyses to identify potential modifying effects on the association between gestational diabetes mellitus and cardiovascular disease mortality by using Cox proportional hazards models 17
Figure S6. Subgroup analyses to identify potential modifying effects on the association between hypertensive disorders of pregnancy and cardiovascular disease mortality by using Cox proportional hazards models 18
Table S5. Sensitivity analyses: history of gestational diabetes mellitus (GDM) and hypertensive disorders of pregnancy (HDPs) and the risk of all-cause mortality and premature mortality after excluding patients who died or lost follow up within 5 years after baseline 19
Table S6. Sensitivity analyses: history of gestational diabetes mellitus (GDM) and hypertensive disorders of pregnancy (HDPs) and the risk of all-cause mortality and premature mortality after excluding patients less than or equal to 60 years old at baseline 20
Table S7. Sensitivity analyses: history of gestational diabetes mellitus (GDM) and hypertensive disorders of pregnancy (HDPs) and the risk of all-cause mortality and premature mortality after further restricting to the pre-pandemic follow-up period 21
Table S8. Comparison of baseline characteristics between participants included and excluded 22

Figure S1. Participants selection diagram

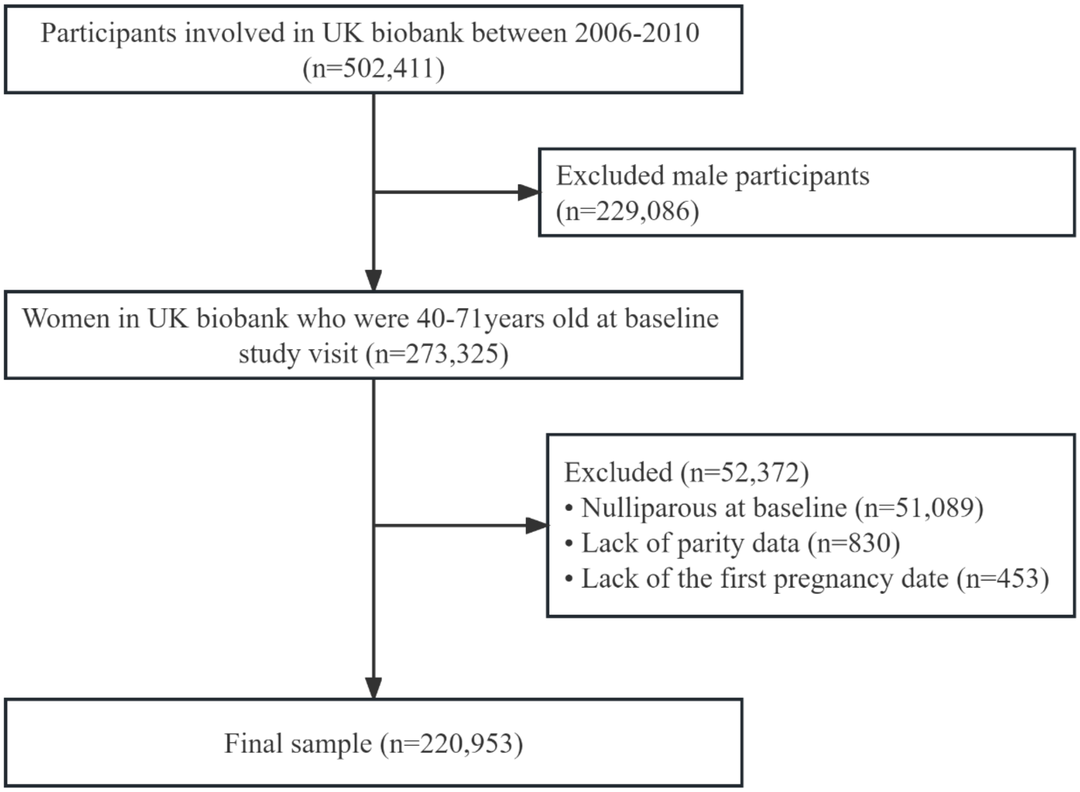

Table S1. Outline of JoGH's Guidelines for Reporting Analyses of Big Data Repositories Open to the Public (GRABDROP) items

1. Please list all papers published by each co-author in previous three years that were based on secondary analysis of a big data repository	The following studies based on UK Biobank data were conducted by co-authors:<br>1 Dai Y, Liu Y, Pan Y, Ma J, Liang J, Zhang W, et al. Associations of cardiovascular health assessed by life's crucial 9 with incident cardiovascular disease and dementia: A prospective cohort study. J Prev Alzheimers Dis. 2025;12:100273.<br>2 Gao D, Zhang W, Zhang Y, Ji M, Han X, Liang J, et al. Reallocation time of accelerometer-measured movement behaviours, genetic susceptibility, and incident chronic obstructive pulmonary disease. Pulmonology. 2026;32:2649099.<br>3 Huang X, Deng S, Xie W, Zheng F. Time in target range of systolic blood pressure and cognitive outcomes in patients with hypertension. J Am Geriatr Soc. 2024;72:423-32.<br>4 Huang X, Gao D, Ji M, Zhang Y, Dai Y, Pan Y, et al. 24-h movement behaviors and the association of chronic pain with venous thromboembolism risk. J Clin Anesth. 2026;112:112227.<br>5 Huang X, Gao D, Zhang W, Ji M, Pan Y, Zhang Y, et al. Sex disparities in the associations between accelerometer-measured movement behaviors and subsequent thromboembolism risk in cancer patients. Biol Sex Differ. 2026;17.<br>6 Huang X, Liang J, Zhang J, Fu J, Chen Y, Xie W, et al. Association of cardiovascular-kidney-metabolic health and apolipoprotein E4 genotype with risk of dementia and mortality. J Alzheimers Dis. 2025;104:1270-80.<br>7 Huang X, Liang J, Zhang J, Fu J, Deng S, Xie W, et al. Association of life's essential 8 with chronic cardiovascular-kidney disorder: a prospective cohort study. BMC Public Health. 2024;24:2448.<br>8 Huang X, Liang J, Zhang J, Fu J, Xie W, Zheng F. Association of cardiovascular-kidney-metabolic health and social connection with the risk of depression and anxiety. Psychol Med. 2024;54:4203-11.<br>9 Huang X, Yang X, Zhu M, Diao X, Zhang J, Pan Y, et al. Magnitude and temporal

	dynamics of dementia risk before and after stroke diagnosis. Int J Stroke. 2025:17474930251397083.<br>10 Huang X, Zhang J, Liang J, Duan Y, Xie W, Zheng F. Association of Cardiovascular Health With Risk of Incident Depression and Anxiety. Am J Geriatr Psychiatry. 2024;32:539-49.<br>11 Li C, Gao D, Cai YS, Liang J, Wang Y, Pan Y, et al. Relationships of Residential Distance to Major Traffic Roads with Dementia Incidence and Brain Structure Measures: Mediation Role of Air Pollution. Health Data Sci. 2023;3:0091.<br>12 Li X, Liang J, Zheng F. Association between hypertension, diabetes, depression, and serum calcium with the risk of all-cause and vascular dementia: findings from the UK biobank. Eur J Nutr. 2024;64:37.<br>13 Li X, Liang J, Zheng F. Sex differences in associations of serum calcium with risk of depression: a prospective cohort study. J Affect Disord. 2026;394:120596.<br>14 Liang J, Li C, Gao D, Ma Q, Wang Y, Pan Y, et al. Association Between Onset Age of Coronary Heart Disease and Incident Dementia: A Prospective Cohort Study. J Am Heart Assoc. 2023;12:e031407.<br>15 Liang J, Li Q, Pan Y, Zhang W, Gao D, Zhang Y, et al. Association Between Age at Diagnosis of Coronary Heart Disease and Incident Depression and Anxiety. Acta Psychiatr Scand. 2025;152:422-31.<br>16 Liang J, Pan Y, Zhang W, Gao D, Ma J, Zhang Y, et al. Associations Between Atherosclerosis and Subsequent Cognitive Decline: A Prospective Cohort Study. J Am Heart Assoc. 2024;13:e036696.<br>17 Liang J, Pan Y, Zhang W, Gao D, Wang Y, Xie W, et al. Associations of age at diagnosis of breast cancer with incident myocardial infarction and heart failure: A prospective cohort study. Elife. 2024;13.<br>18 Liang J, Zhang W, Pan Y, Gao D, Wang Y, Xie W, et al. Associations Between Onset Age of Orthostatic Hypotension and Incident Myocardial Infarction, Stroke, and Dementia: A

Prospective Cohort Study. J Gerontol A Biol Sci Med Sci. 2024;79.
19 Liang J, Zhang Y, Zhang W, Pan Y, Gao D, Ma J, et al. Associations of early-onset coronary heart disease and genetic susceptibility with incident dementia and white matter hyperintensity: A prospective cohort study. J Prev Alzheimers Dis. 2025;12:100041.
20 Pan Y, Liang J, Zhang W, Gao D, Li C, Xie W, et al. Association between Age at Diagnosis of Hyperlipidemia and Subsequent Risk of Dementia. J Am Med Dir Assoc. 2024;25:104960.
21 Pan Y, Zhang W, Dai Y, Liu Y, Gao D, Zhang Y, et al. Association between accelerometer-measured physical activity, genetic risk, and incident type 2 diabetes: A prospective cohort study. Diabetes Obes Metab. 2025;27:7578-86.
22 Wang Y, Gao D, Liang J, Ji M, Zhang Y, Pan Y, et al. Associations of mental and behavioral disorders due to tobacco and alcohol use with incident dementia. J Affect Disord. 2026;401:121239.
23 Wang Y, Li C, Liang J, Gao D, Pan Y, Zhang W, et al. Onset age of diabetes and incident dementia: A prospective cohort study. J Affect Disord. 2023;329:493-9.
24 Yang X, Zhu M, Diao X, Huang X, Pan Y, Dai Y, et al. Dementia Risk in the Decade Before and After Coronary Heart Disease Diagnosis: Hybrid Case-Control and Cohort Study. J Am Heart Assoc. 2026;15:e046596.
25 Zhang W, Liang J, Li C, Gao D, Ma Q, Pan Y, et al. Age at Diagnosis of Atrial Fibrillation and Incident Dementia. JAMA Netw Open. 2023;6:e2342744.
26 Zhang W, Pan Y, Dai Y, Liang J, Ma J, Liu Y, et al. Reallocation of time between accelerometer-derived movement behaviors, genetic susceptibility, and risk of incident dementia, mortality, and premature death: a longitudinal cohort study. Int J Behav Nutr Phys Act. 2025;22:112.
27 Zhang Y, Gao D, Gao Y, Li J, Li C, Pan Y, et al. Gestational diabetes mellitus is associated with greater incidence of dementia during long-term post-partum follow-up. J Intern Med. 2024;295:774-84.

	28 Zhang Y, Gao D, Liang J, Ji M, Zhang W, Pan Y, et al. The role of Life's Crucial 9 in cardiovascular disease incidence and dynamic transitions to dementia. Commun Med (Lond). 2025;5:223.<br>29 Zhang Y, Gao D, Liang J, Ji M, Zhang W, Pan Y, et al. Association between folate deficiency and hypertension: evidence from an observational and Mendelian randomization study. Eur J Prev Cardiol. 2025;32:1310-8.<br>30 Zhang Y, Zhu P, Gao D, Ji M, Pan Y, Dai Y, et al. Joint Associations of Sleep Patterns and Genetic Susceptibility With Dynamic Transitions of Chronic Obstructive Pulmonary Disease and Coronary Heart Disease and the Mediating Role of Inflammatory Biomarkers and Metabolites. J Am Heart Assoc. 2026;15:e046469.<br>31 Zheng F, Liang J, Li C, Ma Q, Pan Y, Zhang W, et al. Age at Onset of Heart Failure and Subsequent Risk of Dementia: A Longitudinal Cohort Study. JACC Heart Fail. 2024;12:826-35.<br>32 Zheng F, Xie W, Li C, Gao D, Liang J. Prediction Abilities of SCORE2 Risk Algorithms for Incident Dementia and All-Cause Mortality: Results From the UK Biobank Cohort Study. J Gerontol A Biol Sci Med Sci. 2023;78:704-10.
2. Please explain the key elements of your study design and the use of the available datasets that make your study an original scientific contribution	This study utilized data from the UK Biobank to investigate the independent and joint associations of GDM and HDPs with long-term all-cause, premature, and cause-specific mortality. To the best of our knowledge, this is the first large-scale population-based cohort study to comprehensively evaluate the separate and combined effects of these two common cardiometabolic pregnancy complications on mortality outcomes. By employing rigorous statistical methods, including multivariable-adjusted Cox proportional hazards models, PAFestimation, and a series of sensitivity analyses, we established robust longitudinal associations. Furthermore, we conducted detailed death attribution analyses to identify the leading causes of death among women with a history of these conditions. Our findings provide novel evidence that the co-occurrence of GDM and HDPs confers a higher mortality risk beyond either condition alone, highlighting the need for integrated postpartum monitoring.

3. Please list all publications that addressed similar research questions in the same dataset and indicate where you cited them in your paper	Three relevant publications were listed as follows:<br>Echouffo Tcheugui JB, Guan J, Fu L, Retnakaran R, Shah BR. Association of Concomitant Gestational Hypertensive Disorders and Gestational Diabetes With Cardiovascular Disease. JAMA Netw Open. 2022;5:e2243618., cited as reference 8 in our paper.<br>Wang YX, Mitsunami M, Manson JE, Gaskins AJ, Rich-Edwards JW, Wang L, et al. Association of Gestational Diabetes With Subsequent Long-Term Risk of Mortality. JAMA Intern Med. 2023., cited as reference 14 in our paper.<br>Pace R, Brazeau AS, Meltzer S, Rahme E, Dasgupta K. Conjoint Associations of Gestational Diabetes and Hypertension With Diabetes, Hypertension, and Cardiovascular Disease in Parents: A Retrospective Cohort Study. Am J Epidemiol. 2017;186:1115-24., cited as reference 18 in our paper.
4. Please explain how you addressed multiple testing through an appropriately rigorous statistical threshold and indicate this in the methods section	Given the prespecified nature of our primary outcomes, we analyzed these outcomes as distinct, a priori hypotheses with clear clinical and biological rationales. We maintained a two-sided significance threshold of $P < 0.05$ for all analyses. While we did not apply a multiple testing correction, we mitigated the risk of false-positive findings through several rigorous approaches: (1) we prespecified a limited number of primary and secondary outcomes based on established literature; (2) we conducted comprehensive sensitivity analyses; and (3) we performed subgroup analyses to test the consistency of associations across different population strata. These additional tests consistently supported our main findings, suggesting that the observed associations are robust and unlikely to be attributable to chance.
5. Please declare to what extent have AI chatbots been used in developing your paper and to which parts of the paper did they contribute	We declare that AI chatbots were not used at any stage in the development of this manuscript.

Table S2. International Classification of Diseases (ICD) codes used for identification of cause-specific mortality*

**Cause of Death**	**ICD-10**
Cancer	C00-C97
Cardiovascular Disease	I00-I99
Respiratory disease	J09-J98
Digestive disease	K20-K93
Neurodegenerative disease	F01-03,G122,G20,G21,G231-233,G238,G239,G30,G31

*Other mortality refers to death from other causes other than cancer, cardiovascular disease, respiratory disease, digestive disease, and neurodegenerative disease.

Table S3. Covariates definition and assessment

Covariates	Definition	Assessment	UK biobank Data-Field ID
Age (years)	Age in years	Difference between date attended baseline assessment and date of birth recorded by NHS	21003
Age at first live birth	Age in years	Touchscreen questionnaire: "How old were you when you had your FIRST child?" and "How old were you when you had your child?" for primiparous women	2754, 3872
Multiple live births	Yes (Number of live births ≥ 5), No (Number of live births < 5)	Touchscreen questionnaire: "How many children have you given birth to? (Please include live births only)"	2734
Race	White, Non-White (Mixed, Asian, Black, Chinese, Other)	Touchscreen questionnaire: “What is your ethnic group?”	21000
Education	Higher education (college or university degree, other professional qualifications), other than higher education	Touchscreen questionnaire: “Which of the following qualifications do you have?”	6138
Current smoking	Yes, No	Touchscreen questionnaire: “Do you smoke tobacco now?” and “In the past, how often have you smoked tobacco?”	20116
Current drinking	At least once per week, less than once per week	Touchscreen questionnaire: “About how often do you drink alcohol?”	1558

Physical activity	Attending moderate or vigorous physical activity 10+ minutes at least twice per week, less than twice per week	Touchscreen questionnaire: "In a typical WEEK, on how many days did you do 10 minutes or more of moderate physical activities like carrying light loads, cycling at normal pace? (Do not include walking);<br>In a typical WEEK, how many days did you do 10 minutes or more of vigorous physical activity? (These are activities that make you sweat or breathe hard such as fast cycling, aerobics, heavy lifting)"	884, 904
Obesity	Yes (BMI ≥ 30 kg/m^2), No (BMI < 30 kg/m^2)	Physical examination: body mass index	21001
Depressed mood	Yes (nearly every day or more than half the days), No (not at all or several days)	Touchscreen questionnaire: "Over the past two weeks, how often have you felt down, depressed or hopeless?"	2050
Hyperlipidemia	Yes, No	Date of the first occurrence of any code mapped to 3-character ICD10 E78. The code corresponds to "disorders of lipoprotein metabolism and other lipidaemias".	130814
Hypertension	Yes, No	Touchscreen questionnaire and verbal interview: self-reported hypertension or anti-hypertensive medication use;	6150, 20002, 6177, 4079, 4080, 93, 94
Diabetes	Yes, No	Touchscreen questionnaire and verbal interview: self-reported diabetes (diabetes, type 1 diabetes or type 2 diabetes) or medication use for lowering blood glucose;<br>Plasma hba1c ⩾ 48 mmol/mol (6.5%)	2443, 20002, 6153, 6177, 30750, 20003

Supplement Methods

Population attributable fraction estimation

We used the “AFcoxph” function provided in the R package “AF” to estimate the population attributable fraction (PAF) function based on a fitted Cox proportional hazard regression model for each of the traffic exposures. The approach estimates the PAF for a time-to-event outcome under the hypothetical scenario where a binary exposure is eliminated from the population. The detailed calculation was summarized below:

$$\text{PAF} = 1 - \frac{\{1 - S_0(t)\}}{\{1 - S(t)\}}$$

The $S_0(t)$ represents the counterfactual survival function for the event if the binary exposure would have been eliminated from the population at baseline, while the $S(t)$ represents the factual survival function. The t represents the time scale variable, e.g. the age in years. The function uses a fitted Cox proportional hazards regression model to estimate the $S(t)$ after adjusting for the covariates included in the model.

Table S4. Baseline characteristics of study participants by gestational diabetes mellitus (GDM) and/or hypertensive disorders of pregnancy (HDPs) status

Characteristics*	Participants				*P* value†
	**GDM−, HDPs− (N=215,689)**	**GDM+, HDPs− (N=1,331)**	**GDM−, HDPs+ (N=3,810)**	**GDM+, HDPs+ (N=123)**	
Age (years)	57.56 (7.81)	52.88 (8.16)	52.09 (8.76)	47.40 (6.42)	<0.001
White ethnic background (%)	203,656 (94.4)	1,117 (83.9)	3,577 (93.9)	101 (82.1)	<0.001
Educational status (%)					
Higher education	93,412 (43.3)	602 (45.2)	1,946 (51.1)	61 (49.6)	<0.001
Age at first live birth (years)	25.86 (5.09)	27.59 (6.08)	28.74 (6.21)	30.67 (6.54)	<0.001
Multiple live births (%)	4,307 (2.0)	60 (4.5)	77 (2.0)	3 (2.4)	<0.001
Follow-up duration (median [IQR], years)	12.87 [12.15–13.57]	12.72 [12.06–13.49]	12.82 [12.20–13.58]	12.61 [11.97–13.19]	<0.001
Mean duration between first birth and enrollment (median [IQR], years)	45.53 [37.29–52.30]	37.12 [29.57–46.62]	35.57 [24.70–46.62]	26.79 [21.74–35.54]	<0.001
BMI (kg/m2)	27.13 (5.08)	28.94 (6.03)	27.98 (5.61)	30.56 (6.79)	<0.001
Obesity (%)	50,760 (23.5)	478 (35.9)	1,115 (29.3)	56 (45.5)	<0.001
Current smoking (%)	18,981 (8.8)	117 (8.8)	247 (6.5)	9 (7.3)	<0.001
Current drinking (%)	133,894 (62.1)	643 (48.3)	2 349 (61.7)	54 (43.9)	<0.001
Physical activity (%)					
keep exercising	167,851 (77.8)	934 (70.2)	2,925 (76.8)	86 (69.9)	<0.001
Depressed mood (%)	11,202 (5.2)	122 (9.2)	203 (5.3)	13 (10.6)	<0.001
Systolic blood pressure (mmHg)	135.82 (19.33)	133.29 (18.33)	139.06 (19.55)	135.54 (16.73)	<0.001
Diastolic blood pressure (mmHg)	80.69 (9.96)	80.44 (10.04)	84.61 (10.25)	84.14 (11.00)	<0.001
Hyperlipidemia (%)	24,002 (11.1)	259 (19.5)	387 (10.2)	21 (17.1)	<0.001
HbA1c (mmol/mol)	35.85 (5.75)	43.71 (14.46)	35.08 (5.91)	44.67 (15.37)	<0.001
Diabetes (%)	8,694 (4.0)	1,113 (83.6)	143 (3.8)	101 (82.1)	<0.001

Hypertension (%)	10,6285 (49.3)	678 (50.9)	2,795 (73.4)	88 (71.5)	<0.001

HDPs – hypertensive disorders of pregnancy, GDM – gestational diabetes mellitus, IQR – interquartile range, BMI – body mass index, HbA1c– hemoglobin A1c.

*The results are presented as n (%) if categorical, mean (standard deviation, [SD]) if normally distributed, and median (interquartile range [IQR]) if nonnormally distributed.

†The differences of baseline characteristics were tested using chi-square test for categorical variables, ANOVA for normally distributed continuous variables and Kruskal-Wallis test for nonnormally distributed continuous variables.

Figure S2. Population attributable fraction of all-cause mortality

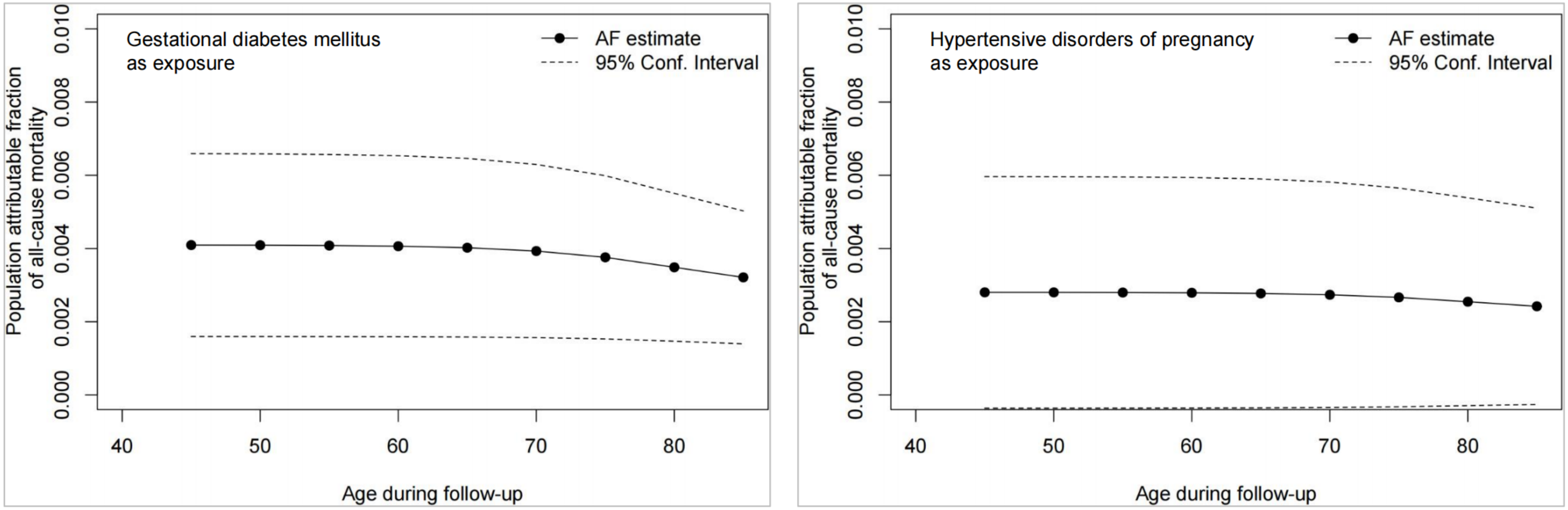

*Fitted Cox regression models with age as the time scale were used to estimate the attributable fraction for each exposure, further controlling for age at first live birth, multiple live births, race, educational status, obesity, current smoking, alcohol intake, physical activity, depressed mood, hyperlipidemia, and hypertension at baseline when gestational diabetes mellitus as exposure; and controlling for age at first live birth, multiple live births, race, educational status, obesity, current smoking, alcohol intake, physical activity, depressed mood, hyperlipidemia, and type 2 diabetes at baseline when hypertensive disorders of pregnancy as exposure.

Figure S3. Population attributable fraction of cardiovascular disease mortality

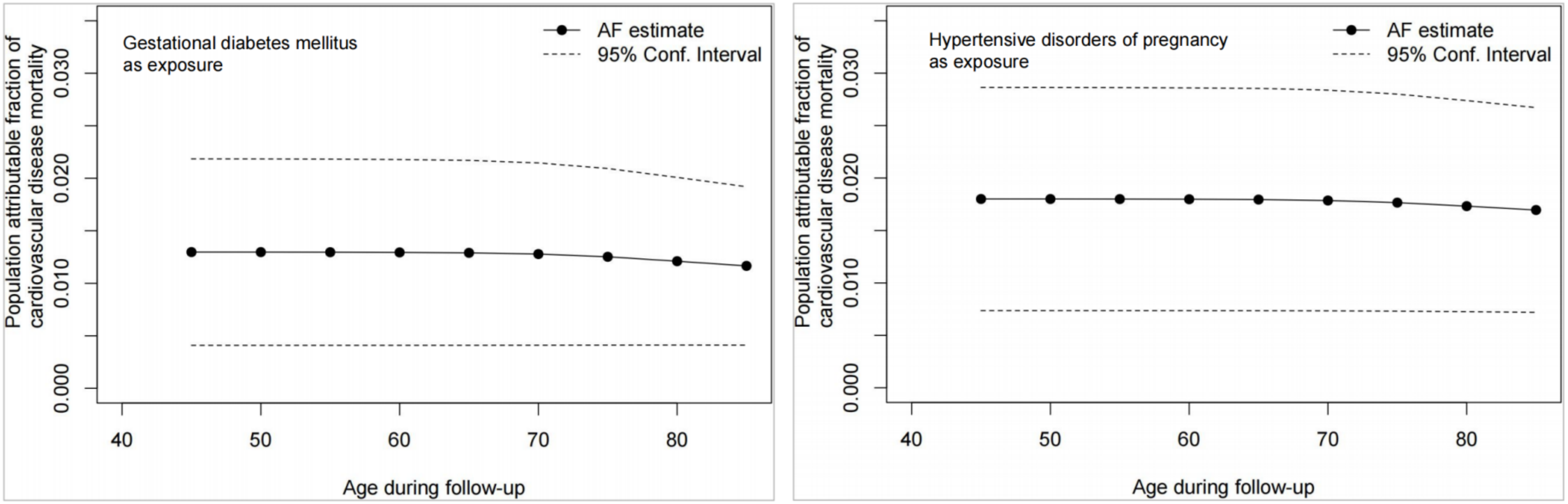

*Fitted Cox regression models with age as the time scale were used to estimate the attributable fraction for each exposure, further controlling for age at first live birth, multiple live births, race, educational status, obesity, current smoking, alcohol intake, physical activity, depressed mood, hyperlipidemia, and hypertension at baseline when gestational diabetes mellitus as exposure; and controlling for age at first live birth, multiple live births, race, educational status, obesity, current smoking, alcohol intake, physical activity, depressed mood, hyperlipidemia, and type 2 diabetes at baseline when hypertensive disorders of pregnancy as exposure.

Figure S4. Subgroup analyses to identify potential modifying effects on the association between gestational diabetes mellitus and all-cause mortality by using Cox proportional hazards models

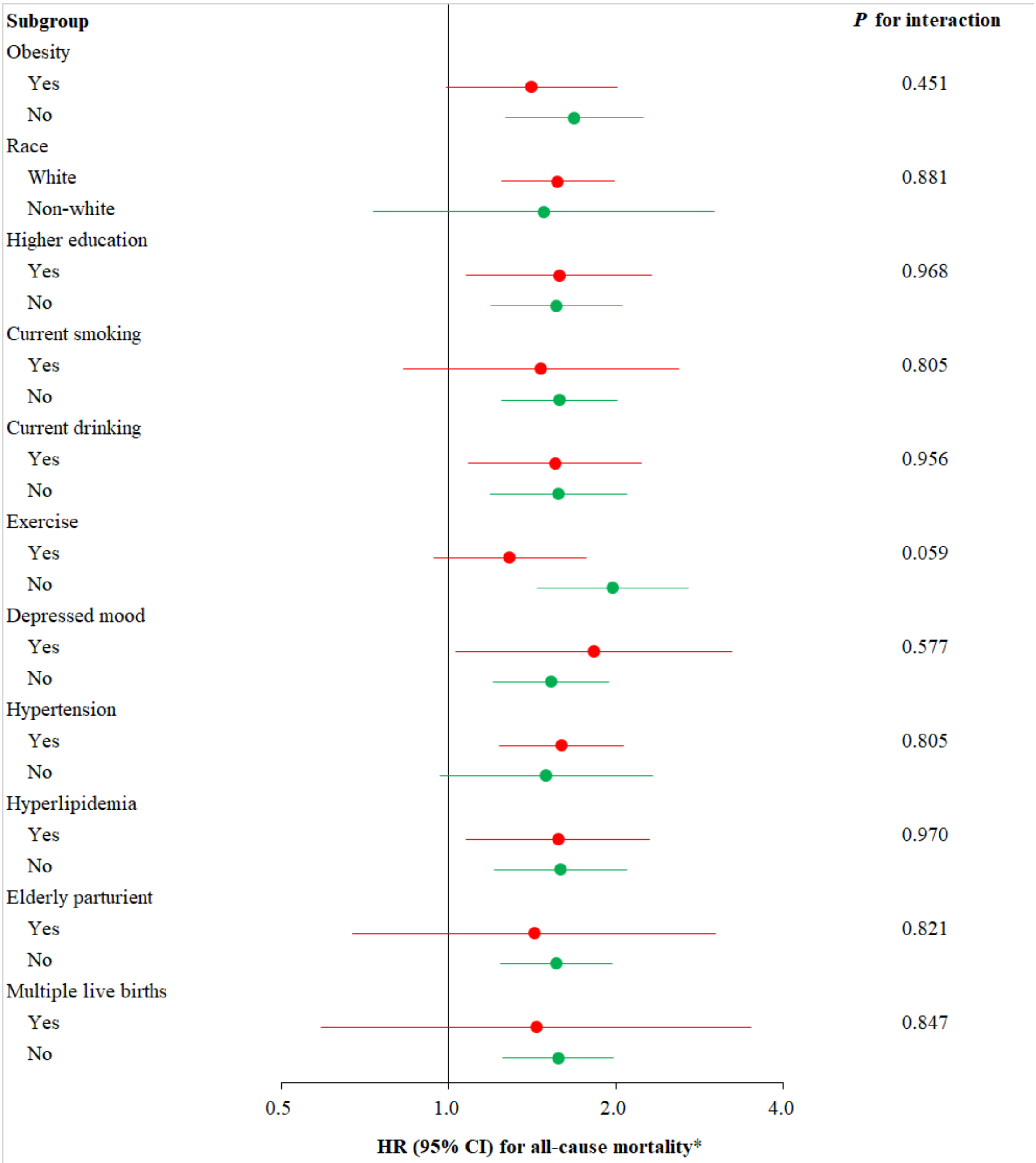

*Adjusted for age at first live birth, multiple live births, race, educational status, obesity, current smoking, alcohol intake, physical activity, depressed mood, hyperlipidemia, and hypertension at baseline, except where an adjusting variable itself was being tested, by using Cox proportional hazards models.

Figure S5. Subgroup analyses to identify potential modifying effects on the association between gestational diabetes mellitus and cardiovascular disease mortality by using Cox proportional hazards models

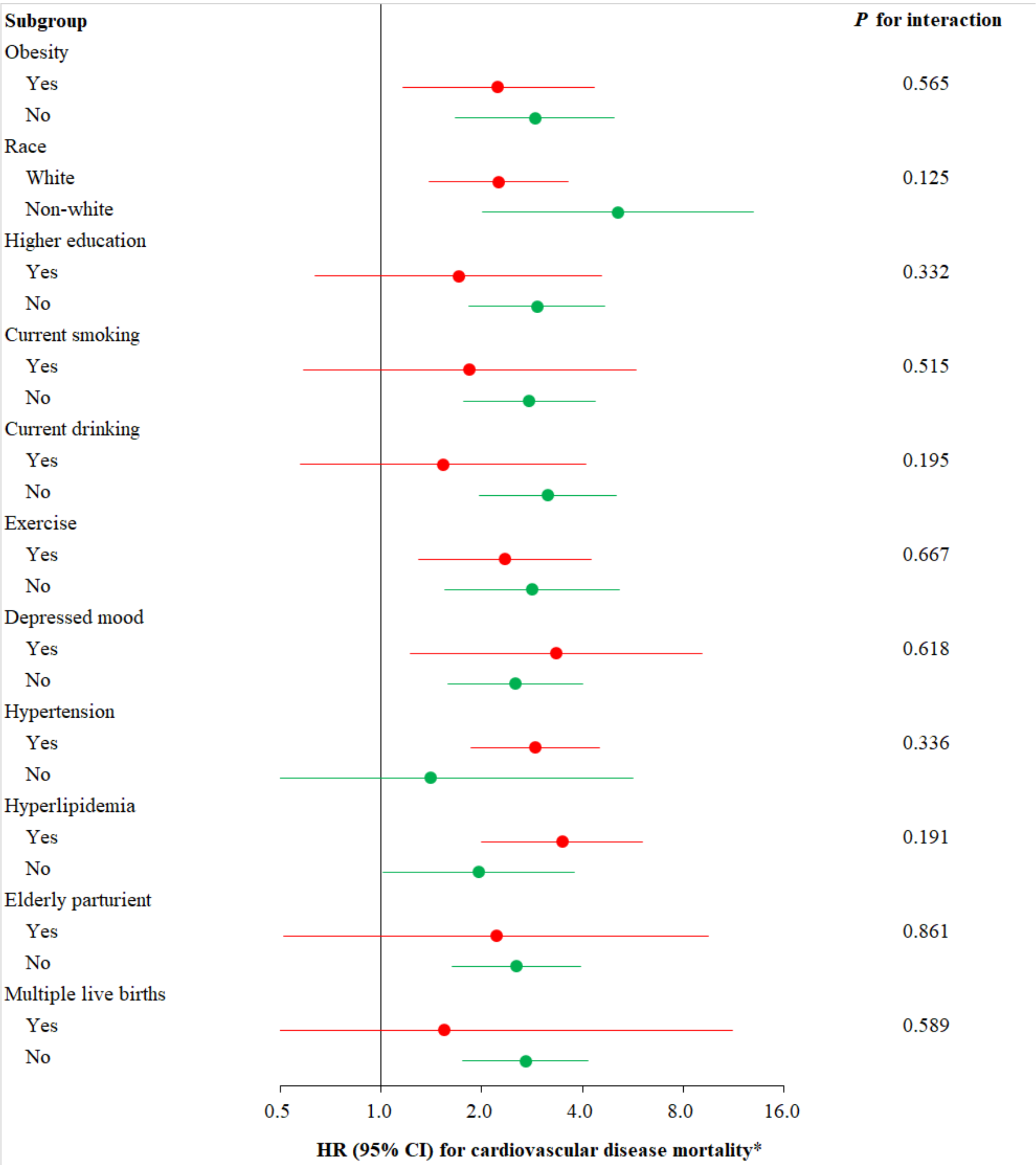

*Adjusted for age at first live birth, multiple live births, race, educational status, obesity, current smoking, alcohol intake, physical activity, depressed mood, hyperlipidemia, and hypertension at baseline, except where an adjusting variable itself was being tested, by using Cox proportional hazards models.

Figure S6. Subgroup analyses to identify potential modifying effects on the association between hypertensive disorders of pregnancy and cardiovascular disease mortality by using Cox proportional hazards models

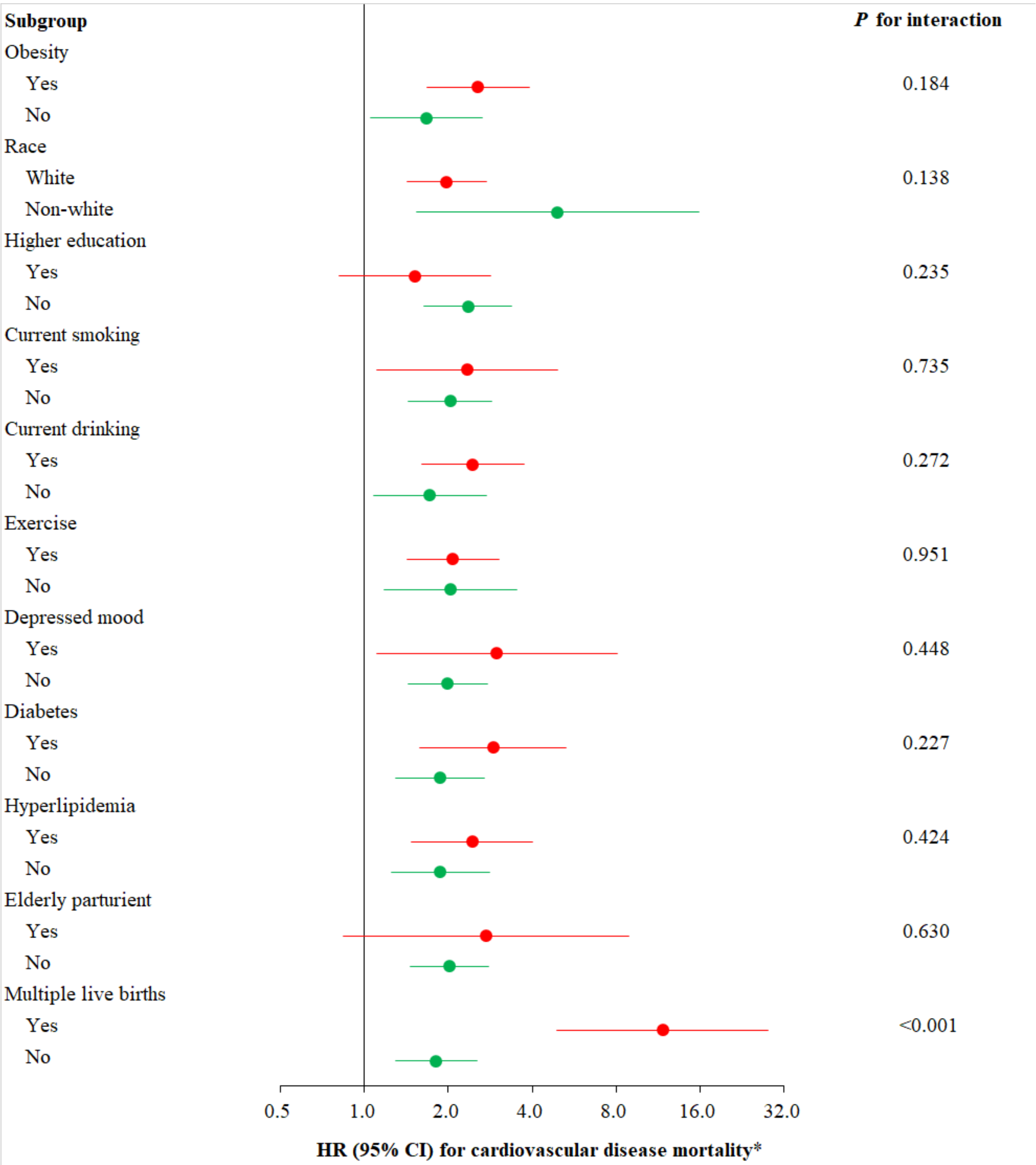

*Adjusted for age at first live birth, multiple live births, race, educational status, obesity, current smoking, alcohol intake, physical activity, depressed mood, hyperlipidemia, and type 2 diabetes at baseline, except where an adjusting variable itself was being tested, by using Cox proportional hazards models.

Table S5. Sensitivity analyses: history of gestational diabetes mellitus (GDM) and hypertensive disorders of pregnancy (HDPs) and the risk of all-cause mortality and premature mortality after excluding patients who died or lost follow up within 5 years after baseline

		Hazard ratio (95% CI)		
**Exposures**	**Events/ Total**	**Unadjusted model**	**Adjusted model 1***	**Adjusted model 2†**
			**All-cause mortality as event**	
No HDPs and no GDM	9,634/212,692	1 [Reference]	1 [Reference]	1 [Reference]
Isolated GDM	52/1,307	1.60 (1.22–2.11)	1.60 (1.22–2.10)	1.43 (1.03–2.00)
Isolated HDPs	124/3,787	1.26 (1.06–1.50)	1.29 (1.08–1.53)	1.25 (1.00–1.56)
HDPs and GDM	6/122	5.71 (2.57–12.72)	6.08 (2.73–13.52)	5.71 (2.38–13.74)
			**Premature mortality as event**	
No HDPs and no GDM	5,976/212,692	1 [Reference]	1 [Reference]	1 [Reference]
Isolated GDM	35/1,307	1.57 (1.13–2.19)	1.57 (1.12–2.19)	1.43 (1.03–2.00)
Isolated HDPs	79/3,787	1.22 (0.97–1.52)	1.24 (1.00–1.55)	1.25 (1.00–1.56)
HDPs and GDM	5/122	6.46 (2.69–15.54)	6.79 (2.83–16.31)	5.71 (2.38–13.74)

HDPs – hypertensive disorders of pregnancy, GDM – gestational diabetes mellitus, CI – confidence interval.

*Model 1: adjusted for age at first live birth, multiple live births, race, and educational status;

†Model 2: model 1 plus obesity, current smoking, alcohol intake, physical activity, depressed mood and hyperlipidemia at baseline.

Table S6. Sensitivity analyses: history of gestational diabetes mellitus (GDM) and hypertensive disorders of pregnancy (HDPs) and the risk of all-cause mortality and premature mortality after excluding patients less than or equal to 60 years old at baseline

		Hazard ratio (95% CI)		
**Exposures**	**Events/ Total**	**Unadjusted model**	**Adjusted model 1***	**Adjusted model 2†**
		**All-cause mortality as event**		
No HDPs and no GDM	9 139/ 102 503	1 [Reference]	1 [Reference]	1 [Reference]
Isolated GDM	48/ 342	1.74 (1.31–2.31)	1.74 (1.31–2.31)	1.58 (1.19–2.10)
Isolated HDPs	96/ 1 025	1.09 (0.89–1.33)	1.11 (0.91–1.36)	1.10 (0.90–1.34)
HDPs and GDM	3/ 9	4.27 (1.38–13.23)	4.63 (1.49, 14.35)	3.60 (1.16–11.14)
		**Premature mortality as event**		
No HDPs and no GDM	5 481/ 102 503	1 [Reference]	1 [Reference]	1 [Reference]
Isolated GDM	31/ 342	1.77 (1.25–2.52)	1.80 (1.26–2.56)	1.65 (1.16–2.34)
Isolated HDPs	51/ 1 025	0.94 (0.71–1.23)	0.95 (0.72–1.25)	0.95 (0.72–1.25)
HDPs and GDM	2/ 9	4.70 (1.18–18.78)	5.10 (1.28–20.30)	4.27 (1.08–16.94)

HDPs – hypertensive disorders of pregnancy, GDM – gestational diabetes mellitus, CI – confidence interval.

*Model 1: adjusted for age at first live birth, multiple live births, race, and educational status;

†Model 2: model 1 plus obesity, current smoking, alcohol intake, physical activity, depressed mood and hyperlipidemia at baseline.

Table S7. Sensitivity analyses: history of gestational diabetes mellitus (GDM) and hypertensive disorders of pregnancy (HDPs) and the risk of all-cause mortality and premature mortality after further restricting to the pre-pandemic follow-up period

		Hazard ratio (95% CI)		
**Exposures**	**Events/ Total**	**Unadjusted model**	**Adjusted model 1***	**Adjusted model 2†**
		**All-cause mortality as event**		
No HDPs and no GDM	9 148/ 215 689	1 [Reference]	1 [Reference]	1 [Reference]
Isolated GDM	58/ 1 331	1.82 (1.41–2.36)	1.82 (1.41–2.36)	1.67 (1.29–2.16)
Isolated HDPs	95/ 3 810	1.01 (0.82–1.23)	1.03 (0.84–1.26)	1.03 (0.84–1.26)
HDPs and GDM	4/ 123	3.62 (1.36–9.60)	3.84 (1.45–10.21)	3.02 (1.13–8.05)
		**Premature mortality as event**		
No HDPs and no GDM	7 270/ 215 689	1 [Reference]	1 [Reference]	1 [Reference]
Isolated GDM	48/ 1 331	1.80 (1.36–2.39)	1.80 (1.36–2.39)	1.66 (1.25–2.20)
Isolated HDPs	76/ 3 810	0.97 (0.78–1.22)	0.99 (0.79–1.25)	1.01 (0.80–1.26)
HDPs and GDM	4/ 123	4.18 (1.58–11.06)	4.38 (1.64–11.67)	3.71 (1.39–9.81)

HDPs – hypertensive disorders of pregnancy, GDM – gestational diabetes mellitus, CI – confidence interval.
*Model 1: adjusted for age at first live birth, multiple live births, race, and educational status;
†Model 2: model 1 plus obesity, current smoking, alcohol intake, physical activity, depressed mood and hyperlipidemia at baseline.

Table S8. Comparison of baseline characteristics between participants included and excluded

Characteristic, No. (%)*	Excluded (n=52 372)	Included (n=220 953)	P for difference†
Age (years)	54.43 (8.2)	57.44 (7.9)	<0.001
White ethnic background (%)	48 693 (93.0)	208 451 (94.3)	<0.001
Educational status (%)			
Higher education	29 463 (56.3)	96 021 (43.5)	<0.001
Age at first live birth (years)	NA	25.92 (5.14)	NA
Multiple live births (%)	53 (11.7)	4 447 (2.0)	<0.001
Follow-up duration (median [IQR], years)	12.8 [12.1, 13.5]	12.9 [12.2, 13.6]	<0.001
Mean duration between first birth and enrollment (median [IQR], years)	NA	45.4 [37.0, 52.2]	NA
BMI (kg/m2)	26.8 (5.6)	27.16 (5.1)	<0.001
Obesity (%)	11 888 (22.7)	52 409 (23.7)	<0.001
Current smoking (%)	5 007 (9.6)	19 354 (8.8)	<0.001
Current drinking (%)	3 2965 (62.9)	136 940 (62.0)	<0.001
Physical activity (%)			
keep exercising	40 035 (76.4)	171 796 (77.8)	<0.001
Depressed mood (%)	2 971 (5.7)	11 540 (5.2)	<0.001
Systolic blood pressure (mmHg)	133.1 (18.7)	135.9 (19.3)	<0.001
Diastolic blood pressure (mmHg)	80.6 (10.2)	80.8 (10.0)	<0.001
Hyperlipidemia (%)	4 560 (8.7)	24 669 (11.2)	<0.001
HbA1c (mmol/mol)	35.45 (6.3)	35.89 (5.9)	<0.001
Type 2 diabetes (%)	2 157 (4.1)	10 051 (4.5)	<0.001
Hypertension (%)	22 788 (43.5)	109 846 (49.7)	<0.001

IQR – interquartile range, BMI – body mass index, HbA1c– hemoglobin A1c.
*The results are presented as mean (SD), median (IQR), or n (%).
†T test used in case of normality, Mann-Whitney U test in case of non-normality of the distribution, chi^2 test used for categorical variables.
SD, standard deviation; IQR, interquartile range; BMI, body mass index; HbA1c, glycated hemoglobin A1c.
